# Quantum Sufficit

**Published:** 1881-02

**Authors:** C. F. Nichols

**Affiliations:** Boston


### QUANTUM SUFFICIT.

BY C. F. NICHOLS, M.D., BOSTON.

"Be thou content to know not, knowing thus,
Thy way of right and duty."

To understand doubters we should remember that all men growing up have seasons of doubting, ignoring, forgetting. "Pity 'tis, 'tis true," that these "cry-baby" times of homœopathic medical men are so often, of late, inflicted upon their "brethren;" for there is much work to be done, and an ample literature is available, to teach and encourage Faint-hearts, Pliables and Ready-to-halts.

Further to understand doubters, yet another matter should be considered. A false modesty exists among doctors, oftenes paraded by the "experienced," who occasionally announce that their wisdom, faithfulness, and medical practice, are grown so great as to have led to their *discovery* that patients get well, independently of medical treatment. This originated in the old school. Abercrombie, Pereira and others having sincerely doubted the efficacy of drugs, it became the fashion to doubt, and an amusing array of skeptics promptly appeared in print and in the lecture-room, displaying such premature wisdom as might gain the respect of colleagues and students. Such self-abnegation digests better than similar disparagement from others.

Doubtless we all like at times to appear *blasé*, and the narrow vanity inherent in skepticism has exerted its usual influence upon the doubters.

Dr. C. Wesselhoeft reports numerous cures by thirtieth and two hundredth potencies, some of which he emphatically attiib utes to his prescriptions;* then he figures at solubles, pretends to test metals,† and decides that the material vehicle (conveying curative power), must be visible—that is, the mould of the statue must not be removed. The mould conceals its own creation, yet it must not be taken away that the statue may be seen. The two hundredth potency *has* cured, yet it cannot cure!‡

Dr. Wesselhoeft can not "invalidate the good results achieved by others," nor "remit a single iota" from the importance of his own cures. "The resources at the command of every physician," diet, regimen and incidental curative agencies, aid the low potentist as well as the high.

Merely to be "*aware* of the arguments which will yet be brought in behalf of the clinical test," does not annul the importance of those arguments. Taken by the horns that clinical bull will throw him; for the doctor says that the value of the clinical test will be greatly enhanced *when construed* according to his own propositions!

The single, final, requisite property of a curative agent is its power to *cure.* Highly potentized metals, chemicals, nosodes, plants, and inert substances have cured in thousands of instances. Their value depends upon such practical tests made as well upon the healthy as the sick. A confirmation of the existence of this singular and special medical power in these high preparations will, we trust, be ultimately vouchsafed by the nat-

---

* *Merc. Viv.* and *Corr.*, *N. E. Gaz.*, 1866, p. 245; 1869, pp. 306, 361. See also *Hahn. Mo.*, 1866, p. 130; *Transac. Amer. Inst.*, 1870; "Raue's Record," 1870–'75, etc., etc.

† "The Effects of Trituration," by C. Wesselhoeft, M.D., Boston. In 1871 (*N.E.Gaz.*, p. 277), Dr. W. remarks, "In most cases the finest chemical tests and even the spectroscope cease to show the presence of a metal after the third or sixth attenuation. Some people would say it had *ceased to be present at all* * * * an empty nothing. Some minds may be satisfied with such arguments, *but they will not stand a single moment before the least effort at reflection.*" (Italics *ours*). Id. p. 528. "The properties of copper * * * recommended by Hahnemann for cholera in 1831," in thirtieth potency and administered in thirtieth potency in Hungary, Austria, Prussia and England. "The result has proved, thousands of times, the correctness of his assertion."

‡ In 1847 Dr. Kidd cured famine-fever in Ireland with medicines moderately attenuated. He fell into tincture-giving in 1878 with no superior results yet published.

ural sciences,* advancing in powers and means of investigation through the development of microscopy, spectroscopy and chemistry.

Let us inquire how much of pure Hahnemannianism has been given up by the doubters and compromisers.

1st. Potentization is opposed, though not completely by all.

2d. Mental symptoms: these are practically ignored. Would it be a long step in the logic of materialism if they were to be altogether blotted out?† True they have helped in the work of saving life and suffering. But the microscopic cap is very loose upon them whilst the pathological cap does not fit at all.

3d. *Similia Similibus Curantur:* This "prejudicial axiom" is attacked by Dr. J. Heber Smith.‡ Dr. McClatchey,§ hesitating to attack, delegates courage to an *imaginary philosophy* not to be found in the sick room. The law of the similars is, however, in great measure supported, apparently as a matter of prudence, until the removal of the last prop shall be in order.

4th. The single remedy. This, of course, is unappreciated.

* "An alcoholic dilution of Aconite, inhaled in all dilutions up to two hundredth dc., may always and with certainty be distinguished from pure alcohol, in comparison with which the highest potency gives an increase of excitability (Osmogram) of from eighteen to thirty per cent. With Thuya four hundred, the increase was forty-four per cent. Two hundredth of Aconite and four hundredth of Thuya always give clearly different neuralanalytic curves. Of the remedies hitherto examined the high, middle and low potencies are readily distinguished from each other. While in many instances the maximum sensitivenesss of an individual was greatest under the middle potencies (fifteen to thirty) it remained in all examined individuals as great in the highest as in the lowest. * * * Neuralanalysis reaches in analytical power far beyond any other known method of investigation, even spectrum analysis. The mathematically constant and most readily observed increase of the physiological action of the drug, developed through potentization, raises Homœopathy to the rank of an exact physiologically based method of cure, in which light its systematic study may justly be established at high schools and universities, and there be presented to the judgment of every man."—"Jaeger, on Neuralanalysis." Abstract from Dr. Korndoerfer's translation, *Hahn. Mo.*, November. Of course (Eds. *Hahn. Mo.*) prudence must be exercised—we suggest Dr. C. Wesselhoeft's taking up Neuralanalysis, confident of fair investigation, and hopeful of a staunch *reconversion.*

† No very serious attempt at this mutilation has been made so far. A *Pharmaco-dynamic Parede* by Rich'd Hughes, L. R. C. P., London, is now acknowledged on all sides to have been intended by its author as a caricature to be read for the entertainment of the moment, since each of its symptomatic indications depends on an appropriate pathological theory changeable from time to time by allopathic authority.

‡ *N. E. Gazette*, March 1874

§ *Hahn. Mo.*, xiii., p. 662. See also Dr. Dake, *Hahn. Mo.* Jan. 1879, *Organon*, i., pp. 56, 195.

One must be familiar with the teachings of Hahnemann's "Organon" before its choice becomes a necessity.

Finally, a few sour arguments are revived from old reviews* —logic of the sort which would prove that Napoleon never lived—and the clinical test is repudiated as evidence. The object for which a physician is supposed to be trained and placed in the community is questioned; reports of diseases relieved are stigmatized as "useless experiments," and ordered to be abandoned, whilst intolerably abusive tirades are bestowed by H. M. Paine upon Hahnemann's personal character.†

Are doubters useful doctors? What do sick people want with them? they are not practical. Drs. S. Potter, C. Wesselhoeft, H. M. Paine, J. E. Smith and E. Guernsey forget, doubt, quibble, and in other highly original ways "test" our medical principles. They indulge in the absurd abuse which greeted the early advocates of homœopathy,‡ and strive to realize chimeras derived from chemical philosophy to be used as proofs to sustain their unnecessary and illogical opposition. Is all this done under the guidance and control of a desire to advance true Homœopathy?

Will a practitioner calling himself homœopathic gain respect from thinking men by prescribing immense mixtures, pills, and salves;§ his prescriptions exhibited by treacherous druggists to the laughter of the contemptuous enemy? Is it not obvious that there are many so-called "leading homœopaths," who are roosting and crowing on fences?

---

* A guide in the labyrinth of these discussions will be found in Dr. Moore's excellent paper, which excepts the views of Mill, Comte, etc., upon the complicated subject of medical statistics, (see pp. 22–26. "Old and New School Therapeutics," F. F. Moore, M.D., Boston, 1880). Note also Prof. Stille's conclusion that statistics in medicine are valueless unless based upon "some sound medical theory."

† *Trans. Am. Inst.*, 1878, *Hom. Times*, Feb. 1879.

‡ At the recent meeting of the American Institute, Dr. E. W. Berridge's advocacy of potentization being known to certain members, these actually voted against permitting that bad man to address the Institute as is elaborately revealed by Dr. Dake in the *Hahnemannian Monthly* for August. Dr. Dake's several stages of anxiety on this score peep thro' "blocks of marble" and bureau drawers marked "no room."

§ See *University Magazine*, and *Liverpool Mercury*, quoted in *Organon*, Oct., 1878.

We have in Boston, (albeit she has rivals in such possessions)* a collection of gems; together they constitute a diadem to deck (?) our city's brow. Let the druggists come forward at the crowning with these mixed prescriptions. They will make a very appropriate offering. On this august occasion commendation may flow from Allopathia, in this wise:† "Well done, sneakingly faithful servant, receive the left hand of our fellowship, your much desired reward—but mind! it means *left*, for no medical society is willing to receive you."

---

### HERING MEMORIAL.

WE are glad to see that steps were taken, at the December meeting of the Phila. County Medical Society, looking to another and larger memorial meeting in honor of the father of American Homœopathy. The meeting held in October last, although due notices were published, was but slimly attended by the physicians of the city and vicinity. Outsiders seemed more anxious to honor the dead hero—the great exponent of true homœopathy—than were his immediate neighbors. This should not be.

We hope the physicians of our city—yea, even those from afar—will inaugurate a fresh memorial offering to the honored dead, and that steps will there be taken not only for a suitable monument for his grave, but also for the publishing of the memorial volume, and for the *correct* completion of his unfinished labors.

All honor is due to Constantine Hering, the noble man, the consistent physician. And let all his brethren show their admiration for their late friend and teacher by heeding his last admonition not to desert *"the strict inductive method of Hahnemann."*

---

* See *The Organon*, i., pp. 829, 442; ii., pp. 8, 161, etc.

† Of course some of those on the fence occasionally hop off and perch behind upon the allopathic car. (Wyld, Pope and others). They are not wanted however (see *London Lancet*, and N. Y. *Hosp. Gazette*, 1879). *Dosis sunt, et quantum sufficit* (*Ap. in altum expulsa et in se cadens* Funklypaths are a dose, whereof "Enough is as good as a feast."

